# Spectral Characteristics of Phase Sensitivity and Discharge Rate of Neurons in the Ascending Tectofugal Visual System

**DOI:** 10.1371/journal.pone.0103557

**Published:** 2014-08-01

**Authors:** Marek Wypych, Attila Nagy, Gabriela Mochol, Andrzej Foik, György Benedek, Wioletta J. Waleszczyk

**Affiliations:** 1 Nencki Institute of Experimental Biology, Warsaw, Poland; 2 The University of Szeged, Szeged, Hungary; University College London, United Kingdom

## Abstract

Drifting gratings can modulate the activity of visual neurons at the temporal frequency of the stimulus. In order to characterize the temporal frequency modulation in the cat’s ascending tectofugal visual system, we recorded the activity of single neurons in the superior colliculus, the suprageniculate nucleus, and the anterior ectosylvian cortex during visual stimulation with drifting sine-wave gratings. In response to such stimuli, neurons in each structure showed an increase in firing rate and/or oscillatory modulated firing at the temporal frequency of the stimulus (phase sensitivity). To obtain a more complete characterization of the neural responses in spatiotemporal frequency domain, we analyzed the mean firing rate and the strength of the oscillatory modulations measured by the standardized Fourier component of the response at the temporal frequency of the stimulus. We show that the spatiotemporal stimulus parameters that elicit maximal oscillations often differ from those that elicit a maximal discharge rate. Furthermore, the temporal modulation and discharge-rate spectral receptive fields often do not overlap, suggesting that the detection range for visual stimuli provided jointly by modulated and unmodulated response components is larger than the range provided by a one response component.

## Introduction

**Figure 1:**
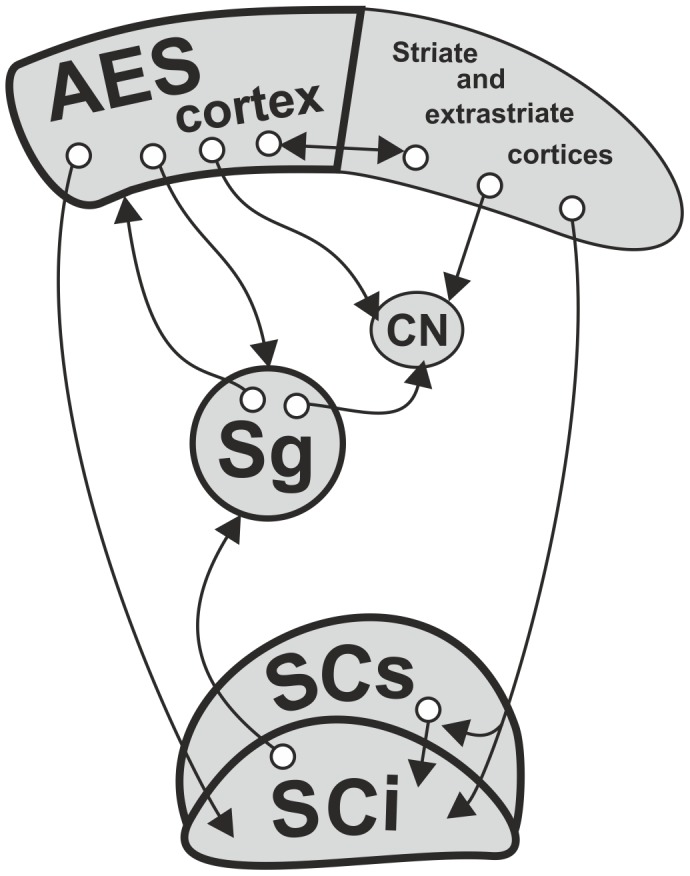
Flow of visual information in the ascending tectofugal system. The simplified schematic diagram shows the afferent and efferent connections of subcortical and cortical structures of the ascending tectofugal visual system. Arrows indicate potential interactions between these structures and the direction of information flow. Structures labeled with bold lettering were examined in the present study. Abbreviations: AES - anterior ectosylvian sulcus, CN – caudate nucleus, SCs, SCi – superficial and intermediate layers of the superior colliculus, respectively, Sg – suprageniculate nucleus of the posterior thalamus.

The ascending tectofugal visual system is considered to be complementary to the geniculo-striate system in the transfer of visual information to the cortex. This extrageniculate tectofugal pathway (Figure 1) originates in the superior colliculus (SC), a midbrain structure, which is the mammalian homologue of the optic tectum of lower vertebrates, and is engaged in spatial localization of visual objects and orienting responses [1], [2]. Several streams of information reach the visual or visual associative cortices from superficial (SCs) and intermediate (SCi) layers of the SC via extrageniculate thalamic nuclei, thus bypassing the dorsal lateral geniculate nucleus (LGN) [3]–[5]. The most important thalamic relay station of the extrageniculate pathway in primates is the pulvinar [6]–[9], while in the feline brain the relay nuclei are composed of the lateral posterior-pulvinar complex (LP-Pul) [10] and the suprageniculate nucleus of the posterior thalamus (Sg) [3], [11].

Geniculo-striate and extrageniculate visual pathways play different roles in vision, which arise from differences in their anatomical connections and neuronal response properties (for rev. see [12]). Many neurons in the retino-geniculo-cortical system, namely those belonging to the parvocellular or X-cell pathway and their projection targets in area 17 (primary visual cortex, striate cortex), are characterized by a preference for relatively high spatial frequencies (SFs) that are suitable for a detailed spatial analysis of the visual image. Neurons in the cat’s SCs, SCi, LP-Pul, and Sg, as well as the cortical targets of the extrageniculate tectofugal pathway, e.g. posteromedial, posterolateral and anteromedial lateral suprasylvian areas (PMLS, PLLS and AMLS), and regions along the anterior ectosylvian sulcus (AES), show a preference for visual stimuli with low spatial frequencies and medium to high temporal frequencies [13]–[20]. Such spatiotemporal spectral response properties suggest that tecto-thalamo-cortical loops of the ascending tectofugal system could be involved in motion perception [14]–[16], [20], [21].

Neurons of the retino-geniculo-cortical pathway respond to drifting gratings with oscillatory modulation in their neuronal firing at the temporal frequency (TF) of the visual stimulus. This issue has been extensively investigated in area 17 (for review see [22], [23]). Oscillatory modulation of responses at the TF of drifting gratings is particularly strong in simple cells - one of two classes of cortical neurons according to the division introduced by Hubel and Wiesel over half a century ago in their seminal papers [24]–[26]. The other class, complex cells, respond to drifting gratings mainly by an increase in their firing rate, with a much lower degree of modulation at the stimulus TF. The strength of firing rate modulation is considered to be a convenient measure of linearity of spatial summation in receptive fields (RFs) of cortical neurons [27], [28], thus simple cells are regarded as highly linear, whereas complex cells have severe nonlinearities. Similarly, in the case of retinal ganglion cells (RGCs), an increase in the unmodulated component of the response to drifting gratings is considered as indicator of nonlinearities [29], [30].

Numerous studies of the retino-geniculo-cortical pathway show that the strength of firing rate modulation at the stimulus TF, varies depending, not only on neuron type, but also on the visual stimulus parameters (e.g. SF and TF of the gratings). Stronger modulations at lower SFs were recorded from area 17 complex and simple cells by Movshon and colleagues [28]. Differences in the spatial frequency-dependence of the modulated response at the stimulus temporal frequency (linear) and unmodulated (nonlinear) response component were reported in earlier RGC studies using gratings [29]–[33]. The dominance of a linear response component at low SFs and a nonlinear response at higher SFs were shown for the Y- [29]–[32], [34] and Q-RGC classes [33].

Oscillations in spike-responses evoked by drifting gratings were also observed in single cell responses recorded from subcortical components of the ascending tectofugal visual system in the cat: i.e. SCs [15], LP-Pul [35] or the Sg, [16], as well as their subcortical and cortical projection targets: the dorsolateral segment of caudate nucleus of the neostriatum (CN) [36], insular cortex [37] and the lateral suprasylvian cortex [18], [19], [38], and area 21b [39]. The number of neurons exhibiting an oscillatory modulation of spike rate and the degree of modulation were found to be much lower in extrageniculate pathway structures compared with area 17 [18], [19], [38]–[40]. This may indicate a much higher content of nonlinearities in the responses of the extrageniculate pathway. That is to be expected due to the two classes of RGCs projecting to the SC (Y and W cells in the cat), which show severe nonlinearities, including an increase of mean firing rate in response to drifting gratings [29]–[33], [41].

In the present study we have asked two questions. Firstly, do neuronal responses in the ascending tectofugal visual pathway show a similar stimulus frequency-dependence in the strength of the modulated response components and mean firing rate compared to that seen in the retino-geniculo-cortical pathway? Secondly, are neuronal response properties related to discharge rates and oscillatory firing modulations, changed or preserved through the tecto-thalamo-cortical circuit? To answer these questions we constructed the spectral spatiotemporal RFs of each recorded neuron for both the mean firing rate and the strength of modulation as measured by a standardized F1 (the Fourier component of the response at the TF of the stimulus) and compared the spectral spatiotemporal RF properties at subsequent stages along the extrageniculate pathway, starting with the SCs, then the SCi, Sg, and the AES cortex.

Preliminary results were presented in abstract form [42].

## Materials and Methods

Extracellular single-unit recordings were performed in the SCs, SCi, Sg of the posterior thalamus, and the cortex along the AES. Data regarding the SF and TF tuning of the SCs, SCi and Sg cells were obtained from previously published studies [12], [15]–[17]. The present study reanalyzed the data in order to describe the organization of the spatiotemporal spectral RFs of single-neurons in the ascending tectofugal visual system. Data obtained from the AES has not been published previously. Detailed information about the number of cells for which responses were taken into account in the present study is shown in Table 1.

**Table 1:** Description of data used in the present study and information of its usage in previous papers.

Brain Structure	Number of animals used	Number of recorded cells included in data analysis	Papers published earlier using the same set of data
SCs	5	63	[12], [15], [40]
SCi	4	99	[17]
Sg	7	105	[16], [40]
AES	4	49	

### Ethics statement

All experimental procedures were carried out to minimize number of animals and their suffering and followed the European Communities Council Directive of 24^th^ November 1986 (86 609 EEC) and the National Institutes of Health guidelines for the care and use of animals for experimental procedures. The experimental protocol was accepted and approved by the Ethics Committee for Animal Research of the University of Szeged.

### Animal preparation, surgical procedures and anesthesia

The experiments were performed on adult cats of either sex, weighing between 2.5–4 kg. The animals were initially anaesthetized with ketamine hydrochloride (Calypsol, 30 mg kg^−1^ i.m.). A subcutaneous injection of atropine sulphate (0.2 ml, 0.1%) was administered preoperatively to reduce salivation and bronchial secretions. After cannulation of the femoral vein and the trachea, the animals were placed in a stereotaxic headholder. All wounds and pressure points were treated with a local anesthetic (1%, procaine hydrochloride). Anesthesia throughout the surgery was maintained by halothane (Halothane, 1.6%) in the inspired air. The animal was immobilized with an initial 2 ml intravenous bolus of gallamine triethiodide (Flaxedil, 20 mg kg^−1^), after which, the animal was artificially ventilated. During recording sessions, a mixture containing gallamine triethiodide (8 mg kg^−1 ^h^−1^), glucose (10 mg kg^−1 ^h^−1^) and dextran (50 mg kg^−1 ^h^−1^; to prevent hypovolemia and brain edema) in a Ringer lactate solution was infused continuously at a rate of 4 ml h^−1^. The eye contralateral to the recording site was treated with atropine sulphate (1–2 drops, 0.1%) and phenylephrine hydrochloride (1–2 drops, 10%) to dilate the pupils, block accommodation, and to retract the nictitating membranes. Contact lenses (+2 diopters) were used to prevent corneal drying and optical correction. The ipsilateral eye was covered during stimulation and recording. Anesthesia was maintained during the recording sessions with a gaseous mixture of air and halothane (0.8–1.0%). The end-tidal halothane value, heart rate (electrocardiogram), and brain activity (electrocorticogram, ECoG; high-amplitude, slow-frequency ECoG activity with sleep spindles) were monitored continuously to ensure an adequate depth of anesthesia. In addition, the animal was checked repeatedly to determine whether any of the experimental procedures or a pinch test to the forepaws induced desynchronization in the ECoG. The minimum alveolar anesthetic concentration (MAC) values calculated from the end-tidal halothane readings were kept in the range (0.9–1.2) as suggested by Villeneuve and Casanova [43]. The end-tidal halothane concentration, MAC value, and peak expired CO_2_ concentration were monitored with a capnometer (Capnomac Ultima, Datex-Ohmeda, Inc.). The peak expired CO_2_ concentration was kept within 3.8–4.2% by adjusting the respiratory rate or volume. O_2_ saturation of the capillary blood was monitored by pulse oxymetry. Body temperature was maintained at 37°C by an automatically controlled warm-water heating blanket. A craniotomy was performed with a dental drill and allowed a vertical approach to the target structures. The dura mater was covered with a 4% warm agar dissolved in Ringer’s solution.

### Extracellular recording of neuronal activity

Vertical penetrations were positioned according to the cat stereotaxic atlas of Snider and Niemer [44]; SCs - Horsley-Clarke co-ordinates A 1–3, L 1–3, depth 15–13; SCi - A 1–3, L 1–3, depth 14–11; Sg - A 4.5–6.5, L 4–6.5, depth 13–10; and AES - A 12–14, L 12–14, depth 17–13. Single-unit recordings were carried out extracellularly via tungsten microelectrodes (AM System Inc., USA; impedance 2–4 MΩ). Recorded signals were conventionally amplified, displayed on an oscilloscope, and transferred to a loudspeaker and PC for on-line analysis and data storage. The visual responsiveness of a neuron was tested and the extent of the visual RF was subjectively estimated by listening to the unit’s response to a moving visual stimulus generated by a hand-held lamp. Single-cell discrimination was performed with a spike-separator system (SPS-8701, Australia), after high-pass filtering of the recorded signal (>500 Hz). At the end of the experiments, the animals were deeply anaesthetized with pentobarbital (200 mg kg^−1^ i.v.) and perfused transcardially with a 4% paraformaldehyde solution. The brains were removed, coronally cut into 50 µm sections, and subsequently stained with Neutral Red (Sigma). Recording sites were localized based on traces of the electrode tracks. The neurons included in the data analyses were located in the SCs, SCi, Sg, and the AES cortex.

### Visual stimulation

Spatiotemporal frequency characteristics of each unit were tested with drifting luminance-modulated sine-wave gratings displayed on a CRT monitor (refresh rate: 80 Hz) positioned at a distance of 42.5 cm from the cat’s eye. Stimuli were presented in a circular aperture with a diameter of 30°, centered either on the centre of the contralateral RF of the recorded unit (SC neurons) or on the *area centralis* (recordings from the AES and Sg). The Michaelson contrast of the grating was held constant at 96%. The mean luminance of the screen was 23 m^−2^ cd. To find the optimal direction of stimulus movement, gratings were moved in eight different directions (0 to 315° in 45° increments). The preferred direction was then used to determine the spatiotemporal frequency characteristics of the cell. The spatiotemporal frequency response profiles were assessed using 24 to 93 frequency combinations of the drifting gratings. The tested SFs ranged from 0.025 to 0.95 cycles degree^−1^ (c/deg), while the TFs varied from 0.07 to 37.24 cycles s^−1^ (Hz). For the purpose of this study, only data obtained during stimulation with drifting gratings at TFs above 2 Hz were used. Each spatiotemporal frequency combination was presented at least 12 times in a pseudo-random sequence with the other seven spatiotemporal combinations. Overall, the presentation of a single stimulus lasted 2 s (for AES recordings this was 3 s), during which the grating remained stationary for the first second. The inter-stimulus interval lasted 0.5 s, during which time a grey screen was shown. Peristimulus time histograms (PSTHs) were constructed on-line to visualize neuronal activity.

### Data analysis

**Figure 2:**
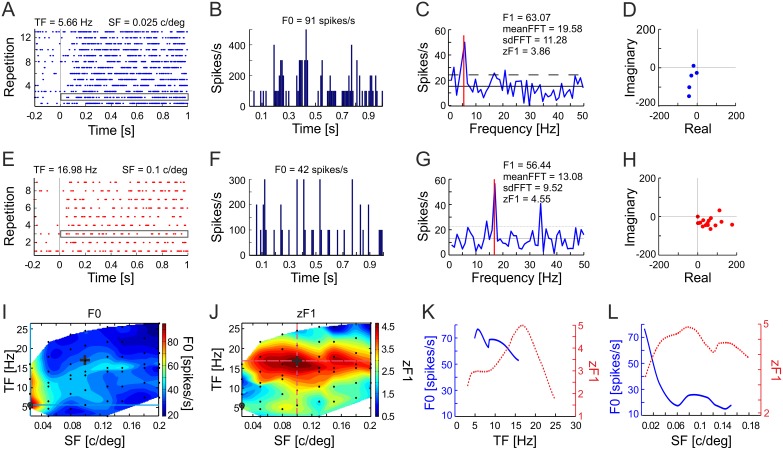
Spectral profile of a Sg cell showing changes in discharge-rate and modulatory responses to sinusoidal drifting gratings. A. Raster plot of cell responses to stimulation with sinusoidal gratings drifting in the optimal direction with spatial and temporal frequency (SF and TF) that elicited a maximum discharge rate (F0). Thin vertical line indicates beginning of grating movement. Single response boxed with grey line was used to construct B, C and D. B. Representative peristimulus time histogram (PSTH) of the unit’s single trial response boxed in A. F0 is defined as the mean firing rate of the response averaged over the time of a single stimulus presentation (1 s of stimulus movement). C. The amplitude spectrum computed from the PSTH shown in B. F1 is the amplitude of the response at the TF of the stimulus (red line). The solid horizontal line depicts mean value of the amplitude spectrum (meanFFT), the dashed line indicates one standard deviation above the mean (sdFFT). zF1 is defined as the ratio of the difference between F1 and meanFFT over the standard deviation from the mean of the spectrum. A value of zF1>1 indicates the presence of modulations. D. Fundamental Fourier components of the neuron’s response to single stimulus trial. Each of the five dots on the complex plane represent fundamental Fourier component of the response to each single, out of five, stimulus cycle of the within one 1 s of grating movement (extracted from single response boxed in A). E–H. Raster plot, PSTH, amplitude spectrum and Fundamental Fourier components of responses to stimulation with a sinusoidal grating drifting in the optimal direction with a spatiotemporal frequency combination that elicited the strongest modulations. Conventions as in A–D. In G notice that zF1, indicating the strength of modulation, is higher than for the response shown in A–D despite a lower firing rate. Dot clustering in the areas distant from the 0 point in D and H indicates the phase sensitivity of the responses. I. Contour plot of the discharge-rate spectral receptive field (RF) of a Sg cell. The surface was fitted to the values of mean discharge rates (F0) obtained in response to grating moving with the SFs and TFs indicated by the black dots. Response strength is color coded according to the scale on the right. Note that the increased firing rate in response to the moving grating is present over a limited range of stimulus SFs and TFs. J. Analogous contour plot of the spectral RF for the same Sg cell constructed by surface fitting of the mean strength of modulations (zF1). Note that the strongest modulations are elicited by stimulus parameters that hardly evoke any increase in firing rate (I). Open circles in I and J represent stimulus parameters evoking highest discharge rate (raster plot of the response is shown in A). The cross indicates stimulus parameters inducing the strongest modulation (raster plot in E). K, L. Temporal (K) and spatial (L) frequency tuning curves obtained from cross-sections of the F0 and zF1 spectral RFs shown in I and J. Blue lines correspond to cross-sections of the discharge-rate RF (I) and the red lines correspond to cross-sections of the zF1 RF (J). Cross-sections were done through the point of the maximum discharge-rate response (marked with circle in I) and through the point of maximum modulation (cross in J).

Off-line data analyses were performed in MATLAB (MathWorks, Inc.). Spike trains were collected in 10 ms PSTH bins (e.g. Figure 2 B, F). Fourier spectra were computed from the PSTHs with a fast Fourier transform (FFT) algorithm, without applying any window function (e.g. Figure 2 C, G). The F1 value (in spikes s^−1^) is defined as the magnitude of the Fourier component at the TF of the stimulus, and the F0 value (in spikes s^−1^) as the mean firing rate of the neuron calculated over the period of stimulus movement.

To identify oscillatory modulation of the firing rate and assess the strength of response modulation to the moving sine-wave grating, standardized F1, or zF1, values were used. In the statistical analysis the standardized value is defined as the difference between the variable and the population mean, divided by the standard deviation of the mean (e.g. [45]). Such data standardization is widely used and is commonly referred to as the ‘z-score’. Previously we showed that zF1 is a reliable measure of the oscillatory modulations in neuronal responses and can be successfully applied to spike train analyses of single visual neurons recorded during stimulation with drifting gratings [40].

The zF1 is defined as the ratio of the F1 component of the response minus the mean value of the amplitude spectrum over the standard deviation of the amplitude values for all frequencies in the spectrum:
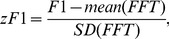
(1)where: zF1 – standardized F1 value, F1 – amplitude component at the TF of the stimulus, mean(FFT) – mean amplitude of the spectrum for frequencies ranging from 1/*T* to *f_psth_*/2 or to the Nyquist frequency, SD(FFT) – standard deviation of the amplitudes in the frequency spectrum for all frequencies from 1/*T* to *f_psth_*/2 (see Figures 2 C and G); *T* in this case is the recording duration of a single response (length of the PSTH) in seconds and *f_psth_ = *1/*T_bin_* where *T_bin_* is the PSTH bin size in seconds. As a result of such standardization, zF1 is essentially independent of the magnitude of the neural discharge and does not depend on the background activity of the analyzed neuron. To calculate zF1, we used the F1, mean (FFT) and SD(FFT) values averaged over trials with identical stimulation parameters. When the F1 value exceeded the mean amplitude of the spectrum by at least one SD(FFT) (zF1≥1), indicating a peak in the spectrum at the TF of the stimulus, the response was considered to be modulated by the grating, otherwise it was considered as non-modulated (zF1<1).

The spatiotemporal frequency or spectral RFs for the neuronal discharge rates and oscillatory modulation (i.e. spectral RFs for F0 and zF1) were constructed as a function of the SFs and TFs of stimulation. A triangle-based cubic interpolation method was used to fit F0 and zF1 surfaces to actual values obtained from experiments (*griddata* MATLAB function; Figure 2 I, J). Optimum SFs and TFs for the discharge rate and zF1, used in further analyses, were determined from the obtained surfaces. The TF and SF tuning curves were obtained from cross-sections of the spectral RFs parallel to the TF and SF axes, respectively. The cross-sections were made through the point giving the maximum discharge rate of the spectral RF or the maximum zF1 value of the modulation RF (Figure 2 K, L). Normalized SF and TF bandwidth was defined as the ratio of the full width of the tuning curve at its half height over the optimum frequency [46].

To assess the variability of modulations we have computed the normalized variance of the Fourier component at the TF of stimulation [47], [48]. Due to a phase shift between stimulus repetitions, we calculated the mean fundamental Fourier component and normalized variance of the Fourier component based on all cycles in a single stimulus repetition (e.g. Figure 2 D, H) and then the variance was averaged across all repetitions of the given stimulus (c.f. [48]):
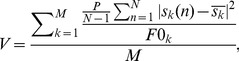
(2)where:



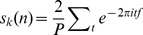
is a complex number representing the fundamental Fourier component of the response to *n*-th cycle of the stimulation with temporal frequency *f* (see Figure 2 D and H); the summation includes spikes occurring at times *t* within the *n*-th cycle of *k*-th stimulus repetition; *P* is the period of one stimulus cycle; *N* is the number of cycles in one stimulus repetition; 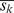 is the mean fundamental Fourier component in the response to *k*-th repetition of stimulus (consisting of N cycles); M is the number of stimulation repetitions with identical SF and TF; and F0*_k_* is the mean response in the *k*-th repetition.

As the variance of Fourier component *s* depends strongly on F0 (see [48]), the variance was divided by F0 for normalization. The units of such defined variability are spikes [48]. In this study, the variance of the fundamental Fourier component in responses maximizing F0 was compared to the variance in responses maximizing zF1.

Statistical comparisons were performed using a non-parametric Wilcoxon matched-pairs signed-ranks test and a Mann-Whitney U test [45]. Statistical significance was accepted for probability (p) ≤0.05 for a two-tailed test. Results are given as the mean ± standard error of the mean (SEM).

The data are available at neurovision.nencki.gov.pl/data/tf_sf_tectofugal_responses.zip.

## Results

Extracellular single unit recordings of responses to sinusoidal drifting gratings over a broad range of SFs (0.025 to 0.95 c/deg) and TFs (2 to 37.24 Hz) were obtained from the SCs, SCi, Sg and AES. All of the neurons responded with changes in the firing rate to visual stimuli within a certain range of SFs and TFs. Within this sample, 32% (20/63) of SCs, 18% (18/99) of SCi, 35% (37/105) of Sg, and 82% (39/49) of AES neurons responded with oscillatory modulations of firing rate at the stimulus TF to at least one spatiotemporal frequency combination.

### Characteristics of spectral receptive fields for F0 and zF1

To examine the firing rate and modulation characteristics of neuronal responses, F0 and the standardized F1, or zF1, were plotted as a function of SFs and TFs of the stimulus, i.e. the spectral RFs for F0 and zF1 were constructed. The discharge-rate spectral RFs provide information about the spatiotemporal properties of the neurons [12], [15], [49]–[53], while F1 spectral RFs bring information about their phase sensitivity [53]. Since F1 depends on the magnitude of the response, we have used standardized F1 values [40] to plot spectral RFs.

Figure 2 shows an example of the spectral characteristics of the discharge-rate and oscillatory modulation of a Sg neuron in response to drifting gratings. The neuron responded with both an increase in firing rate and its modulation at the TF of the stimulus (Figure 2A and E). Representative responses to a single presentation of the stimulus that evoked the maximal discharge rate (F0) and maximal strength of modulation (zF1) are shown in Figure 2B and F, respectively; the corresponding Fourier spectra of the responses are shown in Figure 2C and G. Figure 2I and J show spectral RFs for F0 (I) and for zF1 (J). As can be seen in the contour plots of the spectral maps for F0 and zF1, the region with the highest discharge rate (F0) and the region with the highest modulation (zF1 values) are not overlapping. This means that the spatiotemporal frequency combination that elicited the strongest oscillatory modulation of the discharge rate was not the same as that eliciting the highest mean discharge rate. Interestingly, the strongest modulation was paired with a relatively weak increase in the discharge rate in response to gratings. In this unit, the TF that elicited the strongest modulation was higher than the TF evoking the maximum discharge rate. Moreover, strong firing rate modulations were found for a broad range of stimulus parameters in the SF domain, including those parameters that elicited weak or no changes in mean discharge rate. Such behavior was found in 8% (8 out of 99) of Sg neurons. In contrast, the range of modulatory responses was narrower than the range of discharge-rate responses in the TF domain. Figure 2K and L show TF and SF tuning curves, respectively, for F0 (blue solid lines) and zF1 (red dashed lines) obtained as cross-sections parallel to the ordinate and abscissa of the adequate spectral RFs and through the points of maximum F0 (black circle in I) and zF1 (black cross in J).

**Figure 3:**
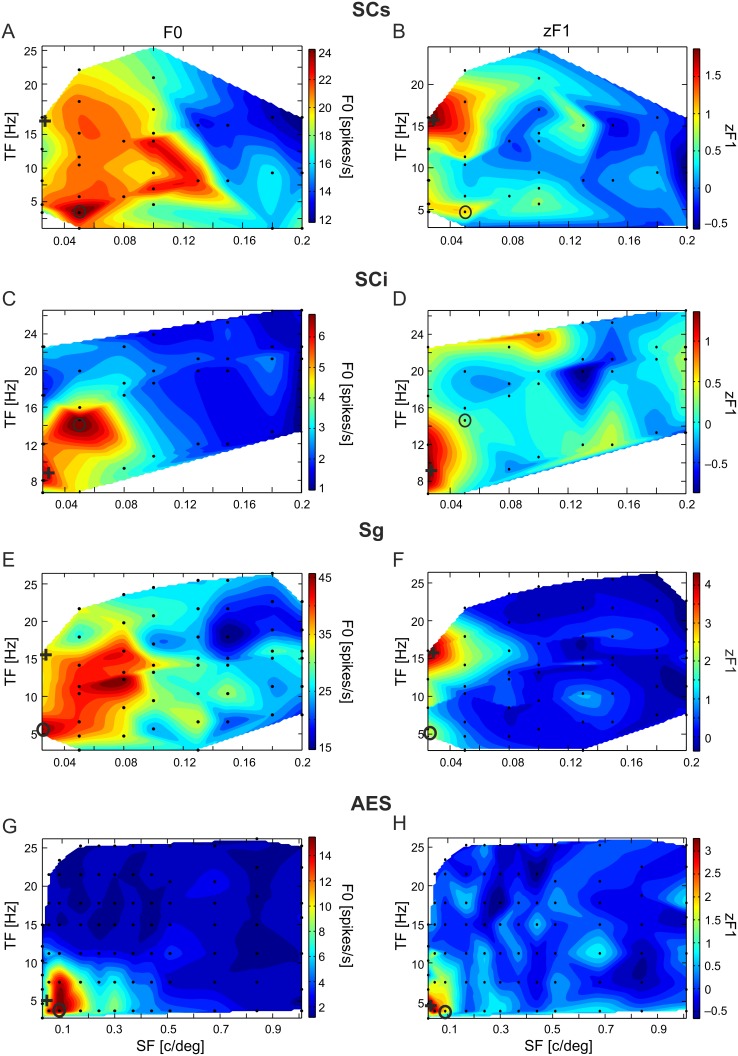
Examples of spectral spatiotemporal RFs for firing rate and strength of modulation. Spectral spatiotemporal RFs computed from firing rate are shown in the left column and those for the strength of modulations determined by zF1 in the right column. The spectral RFs were constructed basing on responses recorded from neurons in the SCs (A, B), SCi (C, D), Sg (E, F) and AES (G, H). Conventions as in Figures 2 I and J.

Figure 3 shows representative examples of the spectral RFs of single neurons recorded from the SCs, SCi, Sg, and the AES, and is analogous to the plots shown in Figure 2I and J. The modulatory response area of the SCs neuron (Figure 3B) is smaller and shifted towards higher TFs compared to the discharge-rate response area in the spectral map for the F0 (Figure 3A). The spectral RF of a SCi neuron (Figure 3C and D) show a shift in TFs and SFs of the maximum zF1 compared with stimulus parameters that evoked a maximum F0; the area of the modulatory response only partially overlaps the discharge-rate response area. In another example of a spectral RF of a Sg neuron (Figure 3E, F; cf. Figure 2), the strongest modulatory response was evoked by a higher TF than that producing the strongest discharge-rate and is similar to the previous examples for Sg and SCs (Figure 2I and J and Figure 3A and B, respectively). However, in contrast to the previous Sg example (Figure 2), the modulatory response area seems to be a subregion of the discharge-rate response area. The representative AES spectral RF (Figure 3G and H) shows that the area of the modulated response is also smaller than the area of the discharge-rate response; only a small shift in the TF can be seen. The highest zF1 value in the SF domain is shifted towards lower frequency values compared with parameters eliciting the strongest discharge-rate.

### Population analysis of spectral receptive fields for F0 and zF1

We also analyzed whether the spatiotemporal properties of the stimulus that elicited the strongest modulations (maximum value of zF1) differed from the stimulus parameters evoking the maximum discharge rate at the population level.

**Figure 4:**
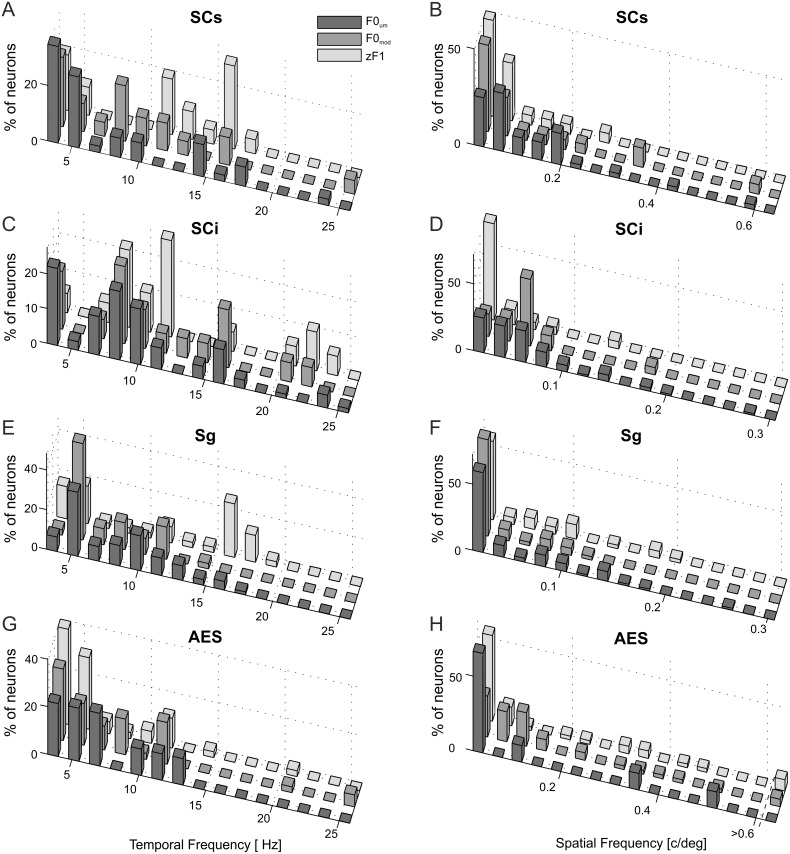
Distributions of temporal and spatial frequencies of gratings that maximized discharge rate and modulation. Shown are distributions of the optimal temporal (left column) and spatial (right column) frequencies of the gratings that evoked maximal discharge rates for neurons with unmodulated (dark grey, R_um_), modulated (medium grey, R_mod_) responses, and those that evoked a maximal zF1 for neurons with modulated responses (light grey, zF1_mod_). Note differences in the range of SF axes for different structures.

**Figure 5:**
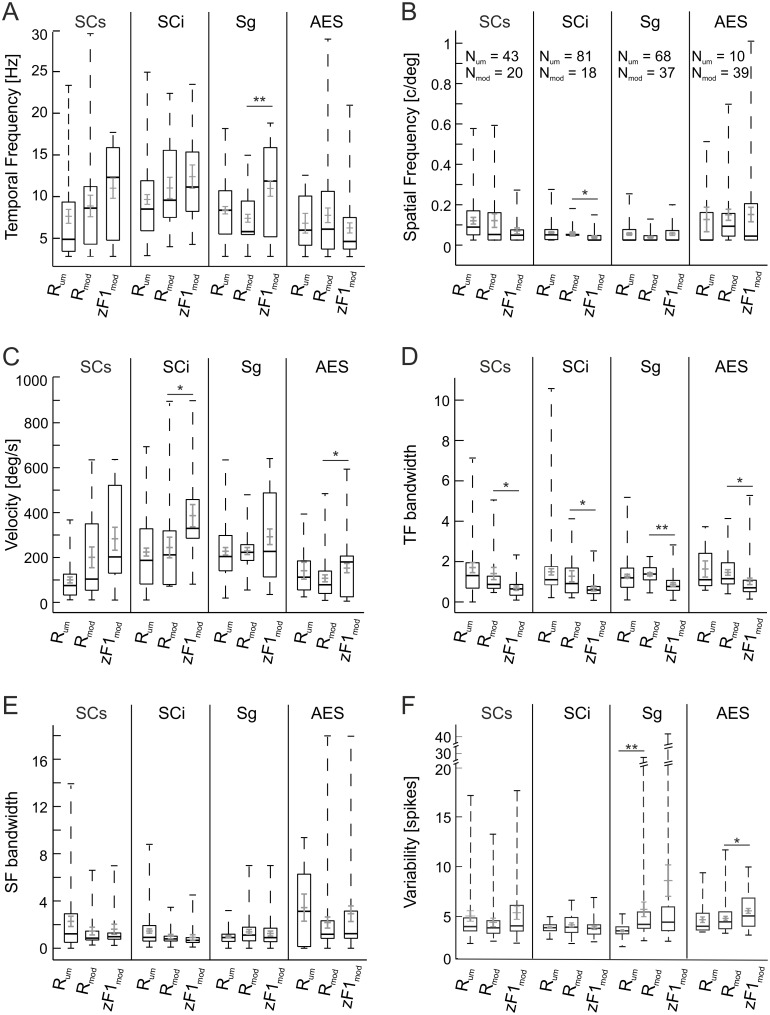
Population analysis of discharge-rate and modulation spectral spatiotemporal RF profiles. Comparison of mean optimal TF (A), optimal SF (B), optimal velocity (C), TF normalized bandwidths (D) SF normalized bandwidths and (E) mean normalized variability of the fundamental Fourier component (F) for discharge-rate responses in subpopulations of neurons with unmodulated (R_um_) and modulated (R_mod_) responses, and for modulations (zF1_mod_) in a subpopulation with modulated responses. Short grey lines with error bars indicate mean **±** SEM. Thick black lines mark median values, boxes cover values from first to third quartiles, and black dashed lines show the ranges of all obtained values. In A, notice that for Sg neurons optimal TF for the modulation component was significantly higher than optimal TF for the discharge rate. In B, the only significant difference in optimal SFs was found between zF1 and the discharge rate in SCi. The optimal stimulus velocity for modulation was significantly higher than the optimal velocity for discharge rate for SCi and AES (C). In D, note that in all structures, the mean TF normalized bandwidth was lower for modulation RFs than for discharge rate RFs (significant in all structures). No significant differences were found for SF normalized bandwidths (E). With the exception of the AES, the normalized variability of the fundamental Fourier component in responses maximizing zF1 did not differ significantly from the variability of responses maximizing F0 (F), suggesting that modulations, if they occur, are stable and do not cause an increase in the variability of the responses. The only significant difference in discharge rates between modulated and unmodulated subpopulations was found in the variability of the fundamental Fourier component in the Sg – this was due to the occurrence of bursts in the activity of modulated Sg neurons.

Figure 4 shows the distribution of optimal TFs (panels A, C, E, and G) and SFs (panels B, D, F, and H) for gratings evoking maximal discharge rates for neurons with unmodulated (dark grey) and modulated (medium grey) responses, and those evoking maximal zF1s for neurons with modulated responses (light grey). Population values and statistical differences between optimal SFs, TFs, and velocities eliciting maximum discharge rates and maximum zF1 values are summarized in Figure 5A–C.

In subcortical structures (SCi, SCs, Sg: Figure 4 panels A, C, and E) TFs eliciting a maximal zF1 tend to be higher than the TFs eliciting a maximal discharge-rate. The clearest differences in optimal TFs for F0 and zF1 were those for Sg modulated neurons (Figure 5A). The mean optimal TF for a maximum discharge rate of these neurons was 7.40±0.47 Hz, while that for the strongest modulation was 11.98±0.94 Hz. The difference was highly significant (p<0.001, Wilcoxon matched-pairs signed-ranks test). In the SCs, the mean optimal TF for a maximum discharge rate and modulation was 8.88±1.29 Hz and 11.01±1.21 Hz, respectively. The difference was not significant (p = 0.20, Wilcoxon test). The mean optimal TF for a maximum discharge rate and modulation in the SCi was 11.04±1.28 Hz and 12.40±1.40 Hz and the difference was also not significant (p = 0.64, Wilcoxon test). In the AES, the mean TF eliciting a maximum zF1 was lower than TFs eliciting a maximum discharge rate (6.25±0.61 Hz vs. 7.76±0.88 Hz), albeit also not significantly (p = 0.26, Wilcoxon test).

The distribution of optimal SFs for the SCs, SCi, Sg and AES neurons is shown in Figure 4 (panels B, D, F and H, respectively). The optimum SFs were similar for each structure. In the SCi (Figure 4D) the maximal modulations were more likely to be evoked by lower SF stimuli (see also Figure 5B). The mean SF for a maximum discharge rate was 0.057±0.008 c/deg, and 0.039±0.007 c/deg for maximum modulation. The difference was significant (p = 0.007, Wilcoxon test). The mean SFs for a maximum discharge rate and maximum modulation in the SCs were 0.12±0.034 c/deg and 0.070±0.015 c/deg respectively, and did not differ significantly (p = 0.28, Wilcoxon test), while in the Sg these were 0.039±0.004 c/deg and 0.055±0.008 c/deg, respectively (also the difference was nonsignificant, p = 0.22, Wilcoxon test). The mean SFs for a maximum discharge rate and modulation in the AES were very similar (0.150±0.028 c/deg and 0.152±0.036 c/deg, respectively; p = 0.49, Wilcoxon test).

The angular velocity evoking maximal modulations was higher than the velocity that elicited a maximum discharge rate in two areas studied (Figure 5C): the SCi (386±49 deg/s vs. 245±46 deg/s; p = 0.013, Wilcoxon test) and AES (153±21 deg/s vs. 107±15 deg/s; p = 0.025, Wilcoxon test). The differences were not significant in Sg (292±35 deg/s vs. 229±16 deg/s; p = 0.43, Wilcoxon test), nor SCs (284±51 deg/s vs. 201±46 deg/s; p = 0.071, Wilcoxon test).

Figure 5 shows comparisons of the temporal (D) and spatial (E) normalized bandwidths for the discharge-rate and zF1 spectral RFs. TF bandwidths calculated from modulation RFs in all structures were significantly lower than those calculated from the discharge-rate spectral RFs. The normalized TF bandwidth in the SCs was 1.41±0.30 for discharge-rate RFs vs. 0.69±0.11 for modulation RFs (p = 0.033, Wilcoxon test) respectively; for the SCi these were 1.27±0.27 vs. 0.67±0.12 (p = 0.033, Wilcoxon test), for the Sg, 1.37±0.08 vs. 0.87±0.09 (p<0.0001, Wilcoxon test), and for the AES, 1.46±0.14 vs. 1.02±0.16 (p = 0.03, Wilcoxon test). In contrast, no significant differences were found for SF bandwidths. In the SCs, the SF normalized bandwidth calculated from the modulation RFs was 1.45±0.33, and 1.61±0.42 (p = 0.68, Wilcoxon test) for the discharge-rate RFs; SCi: 0.99±0.18 vs. 0.92±0.23 (p = 0.23, Wilcoxon test); Sg: 1.39±0.20 vs. 1.25±0.21 (p = 0.51, Wilcoxon test); AES: 2.17±0.47 vs. 2.92±0.66 (p = 0.65, Wilcoxon test).

We also compared the discharge-rate spectral RFs properties between subpopulations of the recorded neurons exhibiting modulated responses and not showing oscillatory modulations of their firing rate. The differences between the optimal stimulus parameters (SF, TF, velocity) evoking a maximum discharge rate in subpopulations of cells with modulated and unmodulated responses were not significant (p>0.08, Mann-Whitney U test; Figure 5A–C). TF and SF bandwidths for units with modulated and unmodulated responses calculated from the discharge-rate spectral RFs were also not significantly different (p>0.12, Mann-Whitney U test; Figure 5D–E).

### Variability of Fourier component at the temporal frequency of the stimulus

To examine the variability of the modulations, the mean normalized variance of the fundamental Fourier component of the response to a single stimulus cycle (see Methods) was calculated for stimulus parameters maximizing zF1 and F0 responses in cells with modulations and for maximizing F0 responses in cells not showing modulations as a control. Besides the difference in variability of the fundamental Fourier component in responses maximizing F0 in subpopulations of Sg cells with modulated and unmodulated responses, and the difference in variability of responses maximizing zF1 and F0 in modulated AES cells (see below), the results of statistical comparisons were not significant (Figure 5F).

In modulated SCs cells, the mean value of the variability of responses with a maximal zF1 was 5.37±0.67 spikes and 4.40±0.43 spikes for maximal F0 responses (difference nonsignificant, p = 0.08, Wilcoxon test). The mean variability for a maximal F0 in the population of cells with unmodulated responses was 5.04±0.53 spikes and did not differ significantly from the value for the subpopulation with modulated responses (p = 0.56, Mann-Whitney U test).

Similarly, in a subpopulation of SCi cells with modulated responses, the variability for responses maximizing zF1 and F0 was 3.86±0.17 spikes and 4.16±0.20 spikes, respectively, and the difference was not significant (p = 0.13, Wilcoxon test). In the population of cells with unmodulated responses, the variability of the fundamental Fourier component was 3.89±0.06 spikes and did not differ significantly from responses maximizing the F0 in the subpopulation with modulated firing (p = 0.53, Mann-Whitney U test).

The variability of responses in a subpopulation of Sg cells with firing rate modulation at stimulus TF was relatively high due to the occurrence of spike bursts from time to time. The mean variability was 8.60±1.58 spikes for responses maximizing zF1 and 5.71±0.74 spikes for responses with a maximal F0 (difference nonsignificant, p = 0.31, Wilcoxon test). However, the variability of responses maximizing F0 for cells with unmodulated firing (3.56±0.08 spikes) was significantly lower than that for the subpopulation with modulations (p<0.0001, Mann-Whitney U test).

In a subpopulation of AES cells with modulated firing rate, the mean variability in responses maximizing zF1 was 5.62±0.25 spikes and significantly higher than responses maximizing F0 (4.86±0.21 spikes, p = 0.018, Wilcoxon test). The variability of responses maximizing F0 in the subpopulation with unmodulated firing was similar (4.73±0.28 spikes, p = 0.37, Mann-Whitney U test).

## Discussion

We have analyzed spatiotemporal response properties of neurons in different structures of the ascending tectofugal system. We focused on the SC – the first stage of the pathway [54], the Sg – a thalamic nucleus relaying tectal information toward associative cortices [4], and the AES cortex – the associative visual cortex, which is a projection target of the Sg [4], [55]. To reveal the characteristics of spectral RFs, we plotted the discharge rates of neurons and the strength of modulation of their firing rate at the TF of the stimulus as a function of the SFs and TFs of the gratings. Characterizing of the spatiotemporal frequency response properties of visual neurons on the basis of the constructed spectral RFs for mean firing rate is a well know and a frequently used approach to describe RFs properties of neurons in optomotor pathway of insects [56], the wallaby nucleus of the optic tract [49], the pretectum and accessory optic system of pigeons [50], [57], [58], cat visual cortex [53], SC [12], [15], V1 and the middle temporal visual area (MT) of macaque monkey [51], [52], and MT of marmoset monkey [59]. In contrast, construction of spectral RFs for the modulated response component is rare [53]. The most noteworthy finding of the present study is that the temporal modulation (zF1) spectral RFs, which reveal phase sensitivity, did not match closely the discharge-rate spectral RFs. Furthermore, in some cells strong modulation was found for spatiotemporal combinations of the stimulus that had a negligible effect on neuronal firing rate. This means that the detection range for visual stimuli provided jointly by modulated and unmodulated response components is larger than the range provided by one component.

### Phase sensitivity in the ascending tectofugal pathway

Oscillations in spike-responses at the TF of drifting sinusoidal gratings have been found not only in the geniculo-striate visual pathway [22], [23], [27], [28], [53], [60], [61], but also in the ascending tectofugal visual system. Earlier studies revealed stimulus-dependent modulations of neuronal activity in the SC [15], [40], [62], LP-Pul complex [35], Sg [16], [40], as well as from the lateral most part of the AES cortex (insular cortex) [37], and the CN [36], [40]. However, to our knowledge, there are no studies that have examined modulatory spectral RFs or compared spatial and temporal frequencies in response to an optimal stimulus in terms of discharge rate versus those maximizing modulations in the extrageniculate visual system.

The standardized F1, or zF1, value was used to estimate the strength of modulation in the recorded responses and, in this way, to determine phase sensitivity of neurons in the extrageniculate visual pathway and AES cortex. We have previously shown that zF1 is a suitable tool for the detection and quantitative assessment of neuronal modulation at the stimulus TF even for neurons with low firing rates and/or a low net magnitude of responses [40]. The proportion of cells exhibiting oscillatory modulations at the TF of the stimulus varied between tectofugal structures. The majority of the AES neurons (82%) showed modulation at stimulus TF, while a smaller proportion showed modulation in the subcortical structures of the ascending tectofugal pathway (32% in the SCs, 18% in SCi, and 35% in Sg). An even smaller proportion of neurons with significant modulatory response components can be assessed when one takes into account only the responses at the maximum discharge rate. Indeed, in an earlier study, analyzing the responses evoking the maximum firing rate, 12% of SCs and 19% of Sg neurons were classified as exhibiting modulatory changes in firing rate in response to visual stimuli, i.e. a zF1>1 [40].

The high proportion of neurons in the AES showing modulatory changes in firing rate and relatively low temporal and high spatial frequencies, which maximize modulations in this area, suggests that modulations in the AES cortex are not derived from the ascending tectofugal pathway. In fact, optimal TFs for modulation of the firing rate of AES neurons are much lower than those of the subcortical structures we examined. Furthermore, comparison of the strength of modulation of neuronal responses in area 17, SCs, and Sg in cats showed a much stronger stimulus phase sensitivity in area 17 compared with the subcortical structures of the extrageniculate pathway [40]. Thus, inputs from lower order visual cortical areas are most likely involved in the stimulus-phase-sensitivity in the AES cortex. A strong firing rate modulation in response to drifting gratings has also been reported for the CN [36], [40]. We observed a large proportion of AES neurons with a modulated responses and postulate that the strong phase-sensitivity of many CN neurons may result from a direct projection from the AES [63].

Unmodulated responses to a drifting grating in the majority of neurons of subcortical structures of the extrageniculate visual pathway most likely result from the prevailing input of W RGCs to the SC, which dominate response characteristics of single neurons in this structure [64]. The highly nonlinear W channel is characterized by a very weak, if any, linear component of responses to drifting gratings [41], [65] and this property of the W channel most likely determines the responses of a majority of neurons in the subcortical extrageniculate pathway. The other reason for a weak linear component in the responses of neurons recorded in our study may lay in the extent of the stimuli used. In our studies, stimuli covered a circular aperture with a diameter of 30°. It has been shown that mean firing rate of most of Y RGCs increases in response to remote gratings of low spatial and high temporal frequencies and this results from a mechanism changing mean firing rate, involving nonlinear subunits, which extend outside the RF center [34]. Intense responses with an unmodulated increase in firing rate to low spatial and high temporal frequency stimuli observed in our study, may result, at least partially, from Y-channel input to the SC, particularly from Y-cell stimulation of the extraclassical RFs.

### Comparison of F0 and zF1 spectral receptive fields in the ascending tectofugal system

Our results showed that the optimum stimulus parameters eliciting the maximum discharge rate in subpopulations of cells with a modulated response component and cells with unmodulated responses were not significantly different. However, there were differences in F0 and zF1 spectral RFs of cells with a modulated response component.

Maximal oscillations could be evoked by stimuli with velocities higher than those evoking maximum discharge rates in two of the areas studied: the Sg and AES. This effect could result from stronger modulation of the firing rate at lower SFs or/and higher TFs of the stimulus compared to stimulus parameters maximizing the discharge rate. Stimulus TFs that maximized modulations in all subcortical structures tended to be higher than TFs maximizing the discharge rate, albeit this was statistically significant only in the Sg. Although there was no difference in SFs at the population level in the SCs and Sg, the difference was evident in responses of many single cells in subcortical structures and was significant for responses recorded from SCi. This is similar to data reported by Movshon and colleagues [28], in which stronger modulations at lower stimulus SFs were recorded from complex and simple cells of area 17. The effect was particularly strong for complex (nonlinear) cells, which showed no or very weak modulation at stimulus parameters evoking the maximal discharge rate [28]. Differences in the spatial frequency-dependence of modulated (linear) and unmodulated (nonlinear) response components to drifting gratings are apparent in Y and Q RGCs [29]–[34], [66], [67]. The dominance of a linear response component at low SFs and a nonlinear response at higher SFs was shown for the Y-RGC class [29]–[32], [34] and Q-RGC class [33]. Similar differences in spatial frequency-dependence of linear and nonlinear response components have also been reported for the Y and non-linear W relay cells of the LGN [65]. Most likely, we could not observe such a strong effect for SFs in the extrageniculate pathway because many of the neurons generate a maximum firing rate at, or close to, the lowest SF of the gratings used in the tests.

The dominance of the linear response component at low SFs or the nonlinear response at higher SFs are typical of Y RGCs [29]–[32]. High TF and low SF stimuli evoking a maximal modulation of the neuronal activity in subcortical structures of the ascending tectofugal pathway (present study) suggest that this phenomenon also depends on the input from the Y cells. The Y channel is characterized by high temporal resolution, good responsiveness at high stimulus velocities, nonlinear spatial summation within the RF, and appears to be involved in the processing of information about fast-moving stimuli. In contrast, X and W cells respond optimally to stimuli moving at low velocities (reviewed in [12], [68]). The spatial resolution of Y-cells is relatively poor, both in the retina [29], [69], and the dorsal LGN [70]–[72]. It has been suggested that Y-type input determines spatiotemporal frequency response properties of a subpopulation of neurons in the superficial retinorecipient layers of the SC [15], [64], as well as their targets in the SCi [17], and neurons in the Sg [16]. This suggests that the SCs Y-channel neurons might be the source of the strong modulatory drive to SCi and Sg, given the discharge rate and preference for relatively high TFs that produce response modulation in a subpopulation of SCs cells (Figure 5A).

To our knowledge, the analyses similar to ours, that is, showing comparisons between the discharge rate and modulation of visual responses over a broad range of spatiotemporal drifting gratings, was conducted for the primary cortex by van Kleef and colleagues [53]. Although detailed comparison of the discharge rate and F1 spectral RFs was not a primary goal of their paper, an example of a simple cell responses (see Figure 2A [53]), demonstrated good overlap between the spectral RFs plotted for F0 and F1 at high contrast. The increase in the F1 spectral RFs plotted at low contrast by van Kleef et al. [53], together with our results, suggests that phase sensitivity improves the visual detection of suboptimal stimuli.

### Role of phase sensitivity in transfer of information in the ascending tectofugal visual pathway

In a subpopulation of Sg neurons, we observed that spatiotemporal frequency combinations that elicited very slight or no change in the net response rate of the neuron, could evoke significant spiking rate modulation at the TF of the stimulus. This suggests that some neurons can transfer information about the stimulus by means of TF modulations of their firing, without any net change in the mean firing rate and pose a question as to whether phase sensitivity participates in the coding of information in the extrageniculate pathway. In subpopulations of cells with modulated responses in the SCs, SCi and Sg the variability of the fundamental Fourier component in the highest modulation was at the level of the variability in response to stimuli inducing a maximum discharge rate. The only significant difference in the variability of responses with a maximal F0 between subpopulations of cells with modulated and unmodulated responses was found in Sg cells, which was accounted for the infrequent occurrence of bursts in the responses of Sg cells showing modulations. Altogether, the values of the normalized variance of the fundamental Fourier component of responses maximizing discharge rate in subpopulations with modulated and unmodulated responses, as well as of responses with the maximal discharge rates and the strongest modulations in cells with modulated responses, tended to be similar. These results can suggest that the variability of the fundamental Fourier component corresponds to the intrinsic variability of the discharge rate, not to the modulation component. We interpret these results as an indication of the stability of modulation and suggest that the modulated responses at the maximum zF1 represent a true communication channel in the extrageniculate visual pathway. Our hypothesis is in line with previous reports in which the temporal organization of spikes was postulated to assure transfer of visual information. It was suggested that bursting-like activity can enhance signal transmission in the visual system as a result of temporal summation of postsynaptic potentials [73]–[76]. In our data set, the effective transfer of modulations seems to be assured as far as the Sg. Also the previously described strong firing rate modulations in the CN [40], the target area of Sg projections, indicate that modulations are passed further. On the other hand, the lack of sensitivity to high TF stimuli in the AEV questions an effective transfer of modulations to the cortical level.

## Conclusions

Spatiotemporal stimulus combinations that elicit maximal firing rate oscillations in neurons of the ascending tectofugal pathway often differ from stimuli that elicit the maximum discharge-rate. Furthermore, the modulation and discharge-rate spectral RFs frequently do not overlap, meaning that the detection range for visual stimuli provided jointly by modulated and unmodulated response components is wider than by one component alone. Maximum modulation was found without a strong increase in the firing rate for some neurons, posing the question as to whether this phenomenon is merely a side effect or whether firing rate modulations take part in coding information about a visual stimulus and constitute a communication channel in the extrageniculate pathway. Our results can not answer this question, however they indicate that in order to fully characterize the spatiotemporal spectral RF properties of neurons exhibiting a modulatory component in their responses, the stimulus evoked modulation of spiking activity should be considered in addition to mean rate, since both rate and temporal changes in firing may take part in coding visual information and assuring its transfer.
